# Clonal dissemination and resistance genes among *Stenotrophomonas maltophilia* in a Greek University Hospital during a four-year period

**DOI:** 10.3934/microbiol.2022021

**Published:** 2022-07-12

**Authors:** Matthaios Papadimitriou-Olivgeris, Fevronia Kolonitsiou, Maria Militsopoulou, Iris Spiliopoulou, Nikolaos Giormezis

**Affiliations:** 1 Division of Infectious Diseases, School of Medicine, University of Patras, Patras, Greece; 2 Infectious Diseases Service, Lausanne University Hospital, Lausanne, Switzerland; 3 Department of Microbiology, School of Medicine, University of Patras, Patras, Greece

**Keywords:** *Stenotrophomonas maltophilia*, resistance, infections, clones, bacteraemia

## Abstract

Treatment of *Stenotrophomonas maltophilia* infections comprises of sulfamethoxazole/tripethoprim (SXT) or fluoroquinolones. We investigated antimicrobial resistance, presence of resistance genes (*sul1*, *smqnr*) and clonal dissemination in *S. maltophilia* from a university hospital. Among 62 isolates, 45 (73%) represented infection. Two isolates (3%) were resistant to SXT and three (5%) to levofloxacin. Twenty-nine isolates (47%), including two out of three levofloxacin-resistant, carried *smqnr*. Resistance of *S. maltophilia* was low and was not associated with *sul1* or *smqnr* carriage. Although high degree of genetic diversity was identified (29 pulsotypes), 22/62 (35.5%) strains were classified into four clones; clone b was associated with bacteraemias.

## 1. Introduction

*Stenotrophomonas maltophilia* is an emerging pathogen which can be found in the environment but is also able to cause infections in immunocompromised patients, critically ill patients and those suffering from cystic fibrosis. Despite its susceptibility to antimicrobials, it has emerged in the last decades as an important nosocomial pathogen with mortality rates between 14 and 69% in bacteraemic patients [1]. Risk factors for infection include prolonged hospitalization, especially in Intensive Care Units, previous antibiotic treatment, chronic respiratory disease, prolonged endotracheal intubation and the presence of a central venous catheter [1],[2].

Sulphamethoxazole/trimethoprim (SXT) remains the treatment of choice for *Stenotrophomonas* infections, whereas, fluoroquinolones are the second-line drug. Treatment can be complicate by the transfer and acquisition of antimicrobial resistance, since mobile genetic elements, such as transposons and plasmids, carry resistance genes [3]. Carriage of *sul1* and *sul2* genes as part on integron I has been associated with resistance to SXT [4]. Another gene family, known as *smqnr* encodes proteins associated with resistance to fluoroquinolones [5].

The aim of this study was to investigate possible clonal dissemination, antimicrobial resistance patterns and the presence of resistance genes among *S. maltophilia* in a Greek University Hospital.

## 2. Materials and methods

The retrospective study was conducted in the University General Hospital of Patras (UGHP), Greece during a four-year period (2014–2017). UGHP is a 800-bed tertiary hospital in Southwestern Greece. The Hospital Ethics Committee approved the study and waived the need for informed consent (HEC No: 785).

All *S. maltophilia* strains isolated from various clinical specimens (blood, bronchial aspirations, intravenous catheters and wounds) were identified to species level by the Vitek 2 Advanced Expert System (bioMerieux, Marcy l'Etoile, France). Infection or colonization was distinguished according to clinical diagnoses. Colonization was defined as the presence of *S. maltophilia* on the respiratory system without causing adverse clinical signs or symptoms and no specific antimicrobial treatment was initiated by the treating physician. Minimum inhibitory concentration (MIC) of SXT was determined by E-test (bioMerieux) and susceptibility against levofloxacin was tested by the disk diffusion method according to CLSI guidelines [6]. Amplification of the resistance genes *sul1, sul2* and *smqnr* was performed by PCRs with specific primers, as published [7].

Strains were classified into pulsotypes by pulsed-field gel electrophoresis (PFGE) of chromosomal DNA *Xba*I digests (Promega Corporation) performed in a CHEF DR III apparatus (Bio-Rad, Richmond, CA). PFGE was performed under the following conditions: initial switch time 5 s, final switch time 5 s, voltage 6V/cm, included angle 120°, run time 23 hours. A dendrogram comparing molecular weights of strains' DNA fragments was performed by FPQuest software version 4.5 (Bio-Rad Laboratories Inc). Patterns differing by less than 79% (corresponding to a difference of less than seven bands) were considered to belong to the same PFGE type [8].

Risk factors for *S. maltophilia* infection as compared to colonization were studied in patients that medical records were available. Epidemiological data, comorbidities, antimicrobial administration, and mortality prediction were obtained from patients' chart reviews.

SPSS version 23.0 (SPSS, Chicago, IL) software was used for data analysis. Categorical variables were analyzed by using the *chi*-square or Fisher exact test. All statistic tests were 2-tailed and *P* < 0.05 was considered statistically significant.

## 3. Results

In total, sixty-two isolates were included (one per-patient) deriving from bloodstream infections (BSIs, n = 26; 42%), surgical site infections (SSIs, n = 13; 21%), catheter-related infections (CRIs, n = 6; 10%) or colonization of the respiratory tract (17; 27%). The majority was recovered from patients hospitalized in medical wards (n = 23; 37%), followed by adult ICU (19; 31%), surgical wards (11; 18%), emergency department (5; 8%) and paediatric ICU (4; 6%).

Two strains (3.2%) were resistant to SXT (MIC: 32 mg/L) and three (4.8%) to levofloxacin. Five strains carried *sul*1 including both SXT-resistant and three SXT-susceptible ones, whereas all isolates were negative for *sul2*. Twenty-nine strains (46.8%), including two out of three levofloxacin-resistant, carried *smqnr*. Four *S. maltophilia* strains carried both *sul*1 and *smqnr* (Table 1). No significant difference among infective and colonizing isolates was identified regarding antimicrobial resistance or genes' carriage.

**Table 1.** Clonal distribution in relation to infection type/colonization and resistant determinants of studied isolates.

Clones	Infection type/ Colonization	SXT-Resistant	Levofloxacin-Resistant	*sul1*	*smqnr*
a (6)	BSI (2)	-	-	-	2
	SSI (2)	-	-	-	2
	Colonization (2)	1	-	1	1

b (6)	BSI (6)	-	-	-	-

c (5)	BSI (1)	-	-	-	-
	SSI (1)	-	-	-	-
	Colonization (3)	-	-	-	3

d (6)	BSI (1)	-	-	-	-
	SSI (1)	-	-	-	1
	CRI (2)	-	-	-	-
	Colonization (2)	-	-	-	2

Others (39)	BSI (16)	-	-	1	6
	SSI (9)	1	2	2	5
	CRI (4)	-	-	-	2
	Colonization (10)	-	1	1	5

Total (62)		2	3	5	29

*Note: Infection or colonization was distinguished according to clinical diagnoses. Colonization was defined as the presence of *S. maltophilia* on the respiratory system without causing adverse clinical signs or symptoms. Number of isolates are presented in parentheses. BSI: Bloodstream infection, SSI: Surgical site infection, CRI: Catheter-related infection.

**Figure 1.**
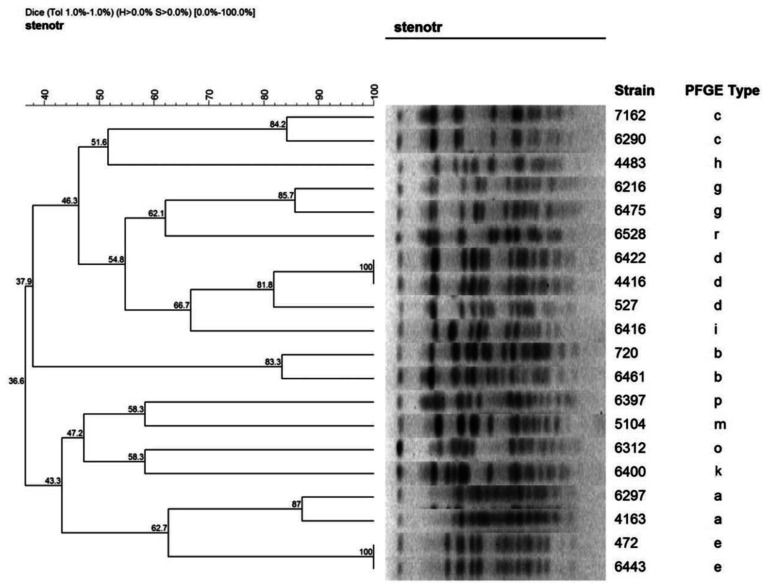
Dendrogram of representative *S. maltophilia* strains.

Twenty-nine pulsotypes were identified by PFGE, with 23 out of 62 (37.1%) strains classified into four main clones, consisting of five or six strains each (Figure 1). The remaining 39 strains were classified into 25 PFGE types, including one or two strains each. No clonal relationship was identified regarding antimicrobial resistance patterns, genes' carriage, or hospital wards. However, a statistically significant association was found for strains of pulsotype b that were exclusively recovered from bacteraemic patients (*P* = 0.004).

Medical records were available for 45 patients (27 infected and 18 colonized). No statistical difference was observed among comorbidities, immunosuppression or presence of resistant genes. Patients with *S. maltophilia* infection were more frequent exposed to ceftazidime/avibactam (48% *vs* 6%; *P* 0.003). No difference in 30-day mortality was observed among patients with infection *vs* colonization (Table 2).

## 4. Discussion

Resistance to the two main antibiotics used for the treatment of *S. maltophilia* infections, SXT and levofloxacin, was low (3.2% and 4.8%, respectively) [9]. In our study presence of the studied resistance genes (*sul*1, *smqnr)* did not confer phenotypic resistance to SXT or levofloxacin, since some phenotypically susceptible strains carried those genes. This finding is in accordance to the literature, since *sul*1 and/or *smqnr* were commonly detected in both resistant and susceptible isolates [7],[10]–[12]. Treatment of infected patients with *S. maltophilia* was successful, even for strains carrying resistance genes. Infective and colonizing isolates were both highly susceptible to SXT and levofloxacin, in accordance to a study by Juhász *et al*, where a low resistance rate was also identified in both groups [13]. Carriage of *sul1* was low (8.06%) and all our strains tested negative for *sul2*, whereas in other studies worldwide both genes have been detected in clinical isolates [14]. *sul2* has even been reported as commoner than *sul1* in one study from 106 strains in India [15].

**Table 2.** Univariate analysis of risk factors for infection by *S. maltophilia* as compared to colonization.

Characteristics	*S. maltophilia* colonization (n = 18)	*S. maltophilia* infection (n = 27)	*P*
Days at risk^a^	36.8 ± 33.9	31.8 ± 21.0	0.613

Demographics			
Age (years)	62.6 ± 13.0	58.2 ± 13.2	0.338
Male gender	10 (63%)	10 (59%)	1.000

Chronic diseases			
Diabetes Mellitus	3 (19%)	1 (6%)	0.335
Chronic Obstructive Pulmonary Disease	0 (0%)	0 (0%)	-
Chronic Heart Failure	1 (6%)	1 (6%)	1.000
Chronic Renal Failure	0 (0%)	3 (18%)	0.227
Malignancy	1 (6%)	2 (12%)	1.000
Immunosuppression	1 (6%)	2 (12%)	1.000

Obesity (BMI ≥ 30kg/m^2^)	3 (19%)	5 (29%)	0.688
Charlson Comorbidity Index	3.8 ± 1.6	3.3 ± 2.5	0.492

Admission data			
APACHE II Score upon admission	20.0 ± 4.4	18.0 ± 4.6	0.271
Prior surgery (prior month)	7 (44%)	9 (53%)	0.732

Antibiotic administration (prior month)			
Penicillin/beta- lactamase inhibitors	9 (56%)	4 (24%)	0.080
3^rd^- and 4^th^-generation cephalosporins	2 (13%)	4 (24%)	0.656
Ceftazidime/avibactam	1 (6%)	13 (48%)	0.003
Carbapenems	13 (81%)	16 (94%)	0.335
Quinolones	2 (13%)	4 (24%)	0.656
Colistin	8 (50%)	14 (82%)	0.071
Aminoglycosides	5 (31%)	9 (53%)	0.296
Tigecycline	4 (25%)	7 (41%)	0.465
Glucopeptides	14 (88%)	10 (59%)	0.118
Linezolid	5 (31%)	7 (41%)	0.721

ICU procedures			
Corticosteroid administration	10 (63%)	13 (77%)	0.465
Parenteral nutrition	8 (50%)	6 (35%)	0.491
Enteral nutrition	9 (56%)	11 (65%)	0.728

Microbiologic data			
Presence of *smqnr* gene (among 21 patients)	5 (56%)	7 (58%)	1.000
Presence of *sul1*gene (among 21 patients)	2 (22%)	3 (25%)	1.000

Outcome			
30-day mortality	7 (44%)	4 (24%)	0.282

*Note: Data are number (%) of patients or mean ± standard deviation. APACHE II: Acute Physiology and Chronic Health Evaluation II. ^a^ Length of stay until infection or colonization.

*S. maltophilia* strains isolated from UGHP patients showed major genetic diversity (29 pulsotypes), as previously reported [9]. This genetic diversity could be attributed to the colonization of patients before or after their admission to the hospital. No correlation between specific clones and resistance to aforementioned antibiotics or carriage of resistance genes was found. The isolation of genetically similar strains, belonging to four main clones, from different patients hospitalized in various wards raised the possibility of transmission within the hospital, but no specific link could be established among personnel or patients' transfer. One of the major clones, pulsotype b, consisted of six strains isolated exclusively from blood infections (*P* = 0.004). Another pulsotype comprised of three isolates derived also from BSIs, but no other statistically significant correlation between clones and infection type was identified. In a previous study of bacteraemias at the UGHP, *S. maltophilia* represented 0.8% of all bloodstream infections, percentage comparable to that from a point prevalence survey of healthcare-associated infections and antimicrobial use in European acute care hospitals (1%) [16],[17]. In another study by Valdezade *et al*., 139 *S. maltophilia* isolates recovered from hospitalized non-cystic fibrosis patients were classified in 99 distinct PFGE profiles, but despite this genetic diversity a few clones were transmitted among different patients causing outbreaks [18].

At the exception of ceftazidime/avibactam, no difference was observed among patients with infection and colonization. The low number of patients might explain the absence of association of typical risk factors, such as immunosuppression, prior carbapenem treatment, and *S. malotphilia* infection [19]. The fact that prior ceftazidime/avibactam use was associated with *S. maltophilia* infection was striking, since ceftazidime/avibactam retains some activity against *S. maltophilia* strains [20].

Our study has limitations since it was performed in a single center, the number of *S. maltophilia* strains was relatively low and PCR was performed only for the main, and not all, genetic determinants conferring resistance to SXT and levofloxacin. Another limitation was the low number of patients for which clinical data were available.

## 5. Conclusions

*S. maltophilia* isolates presented low rates of resistance to SXT or levofloxacin that was not associated with the presence of *sul*1, *sul2* or *smqnr* genes. Even though a high degree of genetic diversity was found, a statistically important correlation of a specific clone with blood infections was detected.
